# Mechanistic insights into the role of microRNAs in cancer: influence of nutrient crosstalk

**DOI:** 10.3389/fgene.2012.00305

**Published:** 2012-12-28

**Authors:** Manasvi S. Shah, Laurie A. Davidson, Robert S. Chapkin

**Affiliations:** ^1^Program in Integrative Nutrition and Complex Diseases, Texas A&M University, College StationTX, USA; ^2^Intercollegiate Faculty of Genetics, Texas A&M University, College StationTX, USA; ^3^Center for Translational Environmental Health Research, Texas A&M University, College StationTX, USA

**Keywords:** microRNAs, cancer, chemoprevention, diet, epigenetics

## Abstract

A plethora of studies have described the disruption of key cellular regulatory mechanisms involving non-coding RNAs, specifically microRNAs (miRNA) from the let-7 family, the miR-17 family, miR-21, miR-143, and the miR-200 family, which contribute to aberrant signaling and tumor formation. Certain environmental factors, such as bioactive dietary agents, e.g., folate, curcumin, polyunsaturated fatty acids, are also thought to impact the progression and severity of cancer. In terms of the chemoprotective mechanisms of action, these bioactive dietary agents appear to act, in part, by modulating tissue levels of miR-16, miR-17 family, miR-26b, miR-106b, and miR-200 family miRNAs and their target genes. However, the mechanisms of nutrient action are not yet fully understood. Therefore, additional characterization of the putative underlying mechanisms is needed to further our understanding of the biology, early diagnosis, prevention, and the treatment of cancer. For the purpose of elucidating the epigenetic landscape of cancer, this review will summarize the key findings from recent studies detailing the effect of bioactive dietary agents on miRNA regulation in cancer.

## INTRODUCTION

MicroRNAs (miRNAs) consist of a diverse class of highly conserved small non-coding RNAs (~22 nucleotides long) shown to play a critical role in basic biological processes such as cellular differentiation, apoptosis, cell proliferation, stem cell development, consequently affecting complex biological events such as carcinogenesis and immune modulation (Esquela-Kerscher and Slack, 2006; Winter et al., 2009). miRNAs are found in both plants and animals and regulate protein expression by acting through perfect or imperfect complementarity to 3^′^ untranslated regions (UTRs) of their “target” mRNAs, which results in repression of target gene expression post-transcriptionally (Esquela-Kerscher and Slack, 2006; Sood et al., 2006). Currently, more than 800 human and mouse miRNAs have been identified (Griffiths-Jones et al., 2008). miRNA studies over the last decade have identified their dysregulation in almost all human malignancies, either acting as oncogenes (oncomirs) or tumor suppressors (Michael et al., 2003; Bandres et al., 2006; Volinia et al., 2006; Yanaihara et al., 2006; Blenkiron et al., 2007; Lee et al., 2007; Porkka et al., 2007; Varnholt, 2008; Bala et al., 2009; Bogner et al., 2009; Chen, 2009; Fassan et al., 2009; Slaby et al., 2009; Zhang et al., 2009; Schaefer et al., 2010; Wang and Sen, 2011; Alhasan et al., 2012; Hu et al., 2012b; Lu et al., 2012; Piepoli et al., 2012).

Bioactive dietary agents appear to have significance in terms of combating pathological diseases including cancer. Indeed, recent evidence indicates that select dietary agents modulate the expression of tumor suppressors/oncogenes involved in signal transduction pathways (Ashendel, 1995; Manson et al., 2000; Neergheen et al., 2010; Shanmugam et al., 2011). Since miRNAs regulate gene/protein expression; there is growing interest in determining the effect of nutritional bioactive agents on the modulation of miRNAs and their target mRNAs in cancer (Davidson et al., 2009b; Shah et al., 2011; Izzotti, 2012; Parasramka et al., 2012a,b). Therefore, this review will focus on the effects of several bioactive dietary treatments in terms of miRNA expression and explain how this might modulate cancer risk.

## BIOGENESIS OF miRNAs

**FIGURE 1:**
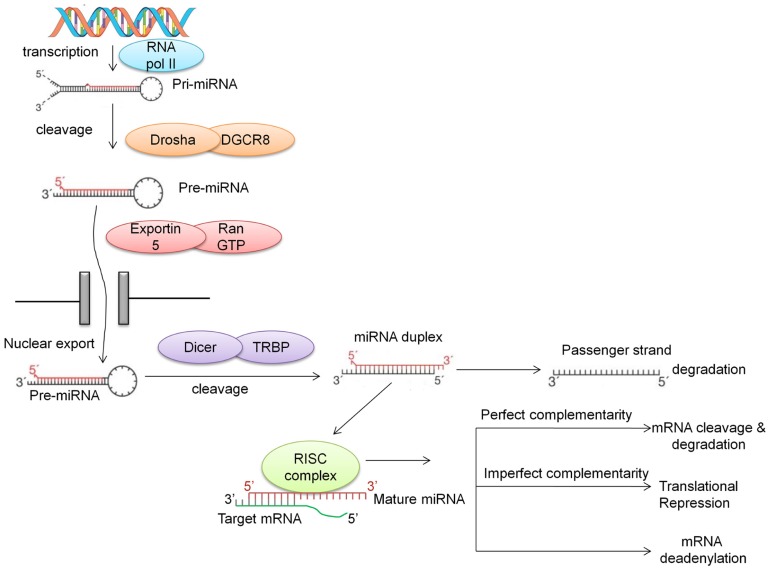
**Biogenesis of miRNA**. miRNAs are first transcribed into pri-miRNA, a hairpin structure which is capped and polyadenylated. Drosha along with DGCR8 cleaves the pri-miRNA into a shorter hairpin structure called pre-miRNA. With the aid of exportin and RanGTP, this pre-miRNA is transported into the cytoplasm, where Dicer cleaves it further to form a miRNA duplex. The main strand of the duplex (the guide strand) is assembled into the RISC complex, while the “passenger” strand is degraded. Recently, this passenger strand has been shown to play a role in targeting mRNAs. The guide strand in the RNA interference silencing complex (RISC; also called the mature miRNA) binds to the 3^′^ UTR of the target mRNA with perfect or imperfect complementarity. This binding either causes mRNA target cleavage or mRNA deadenylation, resulting in translational repression.

MicroRNAs are generally transcribed from intergenic regions, and less so from introns (Ruby et al., 2007). This class of non-coding RNAs is initially transcribed by RNA polymerase II as long hairpin-shaped primary transcripts (pri-miRNAs) that undergo post-transcriptional modifications such as polyadenylation of the 3^′^ end and 7-methyl diguanosine phosphate capping at the 5^′^ end (Cai et al., 2004). The pri-miRNA is then cropped to form a pre-miRNA (~70 nucleotides long) by the enzymatic activity of a cellular RNAse III-type protein endonuclease, Drosha, which together with DGCR8/Pasha protein (DiGeorge syndrome critical region gene) is known as the microprocessor complex (Lee et al., 2003). This pre-miRNA, which has a 2-nt 3^′^ overhang, is recognized by the Ran-GTP-dependent transporter exportin-5 and exported from the nucleus to the cytoplasm (Lee et al., 2003; Lund et al., 2004). In the cytoplasm, the pre-miRNA is then further cleaved by the RNAse III enzyme Dicer which is associated with TRBP (TAR RNA-binding protein) and Argonaute (AGO1-4) to generate a double-stranded (ds) miRNA:miRNA* duplex. This double-stranded duplex is then loaded onto the miRNA associated RNA-induced silencing (RISC) complex and with the aid of AGO proteins is delivered to the target mRNA. The guiding miRNA strand is then unwound by a helicase and is now referred to as “mature” miRNA. This mature miRNA can then hybridize with the 3^′^ UTR of its “target mRNA” with either imperfect or perfect complementarity. Imperfect complementarity leads to translational repression, while binding with high complementarity leads to either cleavage or degradation of the target mRNA (Vasudevan et al., 2007; **Figure 1**). Recent studies have demonstrated that miRNAs may also bind to the 5^′^ UTR and/or the open reading frame (Lytle et al., 2007; Moretti et al., 2010; Qin et al., 2010). Furthermore, there is evidence suggesting that there are alternative pathways for the generation of miRNAs, such as Drosha-independent pathways (Kim, 2005), Dicer-independent pathways (Kawaji et al., 2008; Lee et al., 2009; Cheloufi et al., 2010; Haussecker et al., 2010), and snoRNA-, shRNA- and tRNA-derived pathways (Babiarz and Blelloch, 2008; Ender et al., 2008).

## ROLE OF miRNAs IN CANCER

Recently it has been demonstrated that cancer cells exhibit widespread shortening of 3^′^ UTRs by alternative cleavage and polyadenylation (Mayr and Bartel, 2009). These shorter transcripts produce substantially more protein than their full-length counterparts, in part through escape of miRNA-mediated targeting. The epigenetic nature of this mechanism of oncogene activation directly links miRNAs to cancer risk. As an alternative mechanism, the aberrant expression of miRNAs has been linked to the development of colon (Michael et al., 2003; Bandres et al., 2006; Volinia et al., 2006; Slaby et al., 2009; Piepoli et al., 2012), liver (Varnholt, 2008; Bala et al., 2009; Chen, 2009), lung (Yanaihara et al., 2006; Bogner et al., 2009; Lu et al., 2012), breast (Blenkiron et al., 2007; Fassan et al., 2009; Hu et al., 2012b), prostate (Porkka et al., 2007; Schaefer et al., 2010; Alhasan et al., 2012), and pancreatic cancers (Lee et al., 2007; Zhang et al., 2009; Wang and Sen, 2011; Piepoli et al., 2012). Furthermore, miRNAs have been correlated to tumor location, mutation status of several tumor suppressor genes/oncogenes, and cancer disease stages. For example, in colorectal cancer, miR-31 expression was found to be significantly higher in stage IV tumors as compared to stage II tumors, while miR-21 expression was positively correlated with colorectal cancer metastasis (Slaby et al., 2007). In addition, several miRNAs such as miR-21 have been shown to be aberrantly expressed in almost all types of cancers, while other miRNAs, e.g., let-7, miR-122, are expressed in a highly tissue-specific manner (Castoldi et al., 2011; Iorio and Croce, 2012).

Two key “tumor suppressors,” miR-143 and miR-145, are dysregulated in a number of cancers (Michael et al., 2003; Bandres et al., 2006; Slaby et al., 2007; Xi et al., 2007; Schepeler et al., 2008; Arndt et al., 2009; Motoyama et al., 2009; Earle et al., 2010; Zhu et al., 2011). Functional studies have identified several key targets of miR-143, such as ERK5, KRAS, MAPK7, and DNMT3A, and of miR-145, such as c-MYC, APC, IRS1, STAT1, YES1, and FLI1 (Akao et al., 2006; Arndt et al., 2009; Ng et al., 2009). Moreover, the overexpression of these miRNAs *in vitro* leads to inhibition of cell growth by increasing apoptosis and decreasing cell proliferation (Gregersen et al., 2010; Borralho et al., 2011).

A diverse array of cellular activity has been shown to be modulated by the let-7 family of miRNAs. It has been demonstrated that members of let-7 family act as tumor suppressors or oncogenes based on the tissue type and histological grade of cancer as compared to normal tissue (Johnson et al., 2005; Akao et al., 2006; Sempere et al., 2007; Dahiya et al., 2008; Lawrie et al., 2008; Nam et al., 2008; Ozen et al., 2008; Torrisani et al., 2009; O’Hara et al., 2010). Some of the well-defined targets of the let-7 family are RAS, HMGA2, Blimp-1, and eIF4F (Johnson et al., 2005; Lee and Dutta, 2007; Mathonnet et al., 2007; Mayr et al., 2007; Shell et al., 2007; Nie et al., 2008; Peng et al., 2008; Sun et al., 2009c). Moreover, Ibarra et al. (2007) showed that let-7 is a marker for differentiated cells and is undetected in stem cells.

miR-21 is one of the few well described “oncogenic” miRNAs. High expression of miR-21 has been reported in cancers of the breast (Iorio et al., 2005; Yang et al., 2009; Yan et al., 2011), pancreas (Bloomston et al., 2007; Dillhoff et al., 2008; Moriyama et al., 2009), colon (Asangani et al., 2008; Davidson et al., 2009b; Wang et al., 2009a), and glioblastoma (Chan et al., 2005; Ciafre et al., 2005; Gaur et al., 2011). miR-21 exhibits anti-apoptotic properties by targeting several tumor suppressors such as PTEN, PDCD4, BCL2, TIMP3, TGFβR2, SPRY3, and RECK (Slaby et al., 2007; Schepeler et al., 2008; Wang et al., 2009a; Slattery et al., 2011).

Similar to co-transcribed clusters of genes that code for polypeptides, regions of DNA coding for miRNAs can also occur as polycistronic clusters. One such well-known miRNA cluster, miR-17~92, consists of six individual miRNAs – miR-17, miR-18a, miR-19a, miR-20a, miR-19b-1, and miR-92a (He et al., 2005). These miRNAs are thought to have evolved from two highly conserved mammalian paralogs, miR-106b~25 and miR-106a~363 (Tanzer and Stadler, 2004). Overexpression of this cluster has been observed in several tumor types (He et al., 2005; Volinia et al., 2006; Petrocca et al., 2008). Additionally, miR-17~92 has been shown to suppress c-myc-induced apoptosis in colorectal adenoma and progenitor B cells and thus can be regarded as an oncogene (Diosdado et al., 2009; Li et al., 2012). Using miR-17~92 knockout mice, Ventura et al. (2008) demonstrated that each of the miRNA components in the cluster may have its own specific function in addition to the common functions shared by the entire cluster.

Recently, two miRNA clusters formed from miR-200 family members (the first cluster consisting of miR-200a, miR-200b, and miR-429 and the second cluster consisting of miR-200c and miR-141) have been examined in relation to cancer risk. miRNA profiling studies indicate their down-regulation in breast (Gregory et al., 2008; Radisky, 2011), colon (Burk et al., 2008; Park et al., 2008; Slaby et al., 2009; Mongroo and Rustgi, 2010; Shah et al., 2011), pancreatic (Yu et al., 2010; Soubani et al., 2012), prostate (Kong et al., 2009; Sossey-Alaoui et al., 2009), and other tumor types. miR-200 may exert its effect through a double negative feedback loop between miR-200 family members and transcription factors ZEB1 and ZEB2 (Hurteau et al., 2006; Christoffersen et al., 2007; Burk et al., 2008; Brabletz and Brabletz, 2010). Inhibition of ZEB1 and ZEB2 by these miRNAs is thought to increase key epithelial markers, e.g., E-cadherin, resulting in the acquisition of an “epithelial phenotype” (Christoffersen et al., 2007; Hurteau et al., 2007). Findings from an extensive study performed using NCI-60 cell lines suggest that miR-200 is a marker of epithelial phenotype (Park et al., 2008). Several studies have also linked the miR-200/ZEB system to the TGFβ (Burk et al., 2008; Gregory et al., 2011) and p53 pathways (Chang et al., 2011; Kim et al., 2011; Knouf et al., 2012), which play a role in cancer progression of many tissue types.

In the last few years, there has been a growing interest in determining the biological impact of single-nucleotide polymorphisms (SNP) located in the 3^′^ UTRs of gene targets and in miRNA sequences. This is noteworthy because SNPs in miRNA sequences can influence miRNA processing and/or miRNA–mRNA interactions, thereby modulating cancer risk (Sun et al., 2009a). For example, three SNPs, hsa-miR-196a2 rs11614913 C/T, hsa-miR-499 rs3746444 A/G, and hsa-miR-146a rs2910164 G/C, residing in pre-miRNA regions have been associated with hepatocellular carcinoma (HCC; Xu et al., 2008), familial breast and ovarian cancers (Shen et al., 2008), breast cancer (Hu et al., 2009), prostate cancer (Xu et al., 2010), papillary thyroid carcinoma (Jazdzewski et al., 2008), cervical squamous cell carcinoma (Zhou et al., 2011), gastric cancer (Peng et al., 2010; Zeng et al., 2010), and lung cancer (Tian et al., 2009). Moreover, rs11614913 located within pre-miR-196a2 has been associated with increased risk of lung cancer (Kim et al., 2010). Furthermore, a recent study showed that presence of SNP rs4938723 in the promoter region of pri-miR-34b/c was significantly associated with increased risk of HCC (Xu et al., 2011). Also, the presence of SNPs residing within the 3^′^ UTR of genes that are either tumor suppressors or oncogenes could contribute to tumorigenesis and thus increase the risk of developing cancer (Chin et al., 2008). Studies in breast cancers indicate the presence of SNPs rs799917 in exon (1) of BRAC1 and rs334348 in the 3^′^ UTR of TGFBR1 which are localized to the predicted binding sites of miR-638. These SNPs were associated with increased risk of sporadic and familial breast cancer (Kontorovich et al., 2010; Nicoloso et al., 2010). Additionally, the presence of SNPs in the let-7 complementary sites in KRAS (rs712) were associated with increased risk of both lung and colon cancer (Chin et al., 2008; Landi et al., 2008, 2012). These findings stress the importance of genetic variation in modulating the actions of miRNAs and their target genes.

## MODULATION OF miRNAs BY BIOACTIVE DIETARY AGENTS

There is clinical, experimental, and epidemiological evidence suggesting that diet is one of the most important modifiable determinants of risk for developing a number of chronic diseases. Various natural dietary chemoprotective agents have been shown to exert pleiotropic actions in cancer cells. Recent data suggest that environmental agents, specifically bioactive food components and exercise, play a role directly or indirectly in the modulation of miRNA expression (Davis and Ross, 2008; Davidson et al., 2009b; Saini et al., 2010; Shah et al., 2011; Parasramka et al., 2012b). Observations and mechanisms by which several of the above mentioned dietary factors modulate miRNA expression and function – leading to inhibition of cancer growth, induction of apoptosis and other protective processes – are highlighted below.

### MODULATION OF miRNAs BY FATTY ACIDS

A growing body of evidence demonstrates that high intake of n-3 polyunsaturated fatty acids (PUFAs) suppresses the development of colon cancer (Chang et al., 1998; Davidson et al., 2009a; Kachroo et al., 2011; Turk et al., 2012), breast cancer (Dimri et al., 2010), and glioblastoma (Leaver et al., 2002), by modifying gene expression and cellular signaling pathways. This is consistent with human studies where diets rich in n-3 PUFAs (docosahexaenoic acid, DHA and eicosapentaenoic acid, EPA) found in fish oil, were protective against colon tumorigenesis (Potter, 1993; Chang et al., 1998; Hall et al., 2008; West et al., 2010). In contrast, diets rich in n-6 PUFAs (linoleic acid, LA and arachidonic acid, AA) found in vegetable oils and red meat, enhance both the initiation and promotion of colon cancer (Reddy et al., 1991; Whelan and McEntee, 2004). However the mechanism of action of these fatty acids with respect to the prevention of cancer has not yet been fully established. Therefore, we investigated the chemoprotective effects of n-3 and n-6 PUFAs in a colon carcinogenesis rodent model system and demonstrated that expression of let-7d, miR-15b, miR-107, miR-191, and miR-324-5p were modulated in rats injected with azoxymethane (AOM, a colon carcinogen) fed a fish oil (containing n-3 PUFA) enriched diet (Davidson et al., 2009b). Furthermore, an integrated global approach was used to elucidate the biological effects of these PUFAs in the presence of a carcinogen. Specifically, complementary computational analyses with miRNA and mRNA expression datasets were performed. We observed that a corn oil-cellulose-based diet in the presence of carcinogen compared to fish oil-pectin-based diet increased the expression of miR-16, miR-19b, miR-21, miR-26b, miR-27b, miR-93, 200c, and miR-203, while reducing the expression of some of their targets, e.g., PTK2B, TCF4, PDE4B, HDAC4, and IGF1. These data suggest that dietary PUFAs modulate non-coding RNAs in the colon. In comparison, in glioblastoma cells, following treatment with three different types of PUFAs (GLA, AA, and DHA), several miRNAs including miR-16, miR-143, miR-22, miR-20b, miR-31, miR-145, miR-182, miR-183, miR-200c, miR-26a, miR-206, miR-140, miR-17, miR-29c, and miR-34 were differentially expressed. Specifically, in PUFA-treated cells, miR-143 was reduced, while miR-20b was elevated when compared to untreated cells (Farago et al., 2011). Vinciguerra et al. (2009) observed that unsaturated fatty acids (oleic, palmitoleic, and linoleic acid) reduced PTEN expression in hepatocytes. They reported that treatment with oleic acid (n-9 monounsaturated fatty acids) also up-regulated miR-21 synthesis by activating the miR-21 promoter via an mTOR/NF-κB65-dependent mechanism. *In vitro* studies in breast cancer cell lines (MCF-7 and MDA-MB-231) showed that DHA inhibited the expression of CSF-1 (colony stimulating factor-1). Additionally, DHA treatment inhibited miR-21, which was associated with increased PTEN protein levels and attenuated CSF-1 expression. These results were recapitulated in mouse breast tumor cells (Mandal et al., 2012). Thus, it appears that miRNAs may be involved in mediating some of the anti-oncogenic and chemoprotective properties of PUFAs.

### MODULATION OF miRNAs BY BUTYRATE

Butyrate, a short-chain fatty acid produced via fermentation of dietary fiber predominantly in the distal intestine, is a putative chemoprotective agent. With respect to epigenetic changes, butyrate acts as a histone deacetylase inhibitor capable of decreasing proliferation and increasing apoptosis in colorectal cancer cells (Hodin et al., 1996; Hinnebusch et al., 2002; Chirakkal et al., 2006; Comalada et al., 2006). Studies have demonstrated that these effects are mediated in part through induction of p21waf1/cip1 expression (Crim et al., 2008). Recent evidence suggests that the protective effects of butyrate may be mediated in part by modulating miRNA expression. Hu et al. (2011) showed that upon treatment of human colon cancer cells (HCT116) with butyrate, expression of multiple members of the miR-17~92, miR~18b-106a, and miR-106b~25 clusters were significantly reduced. Also, p21 was determined to be a direct miR-106b target. These data indicate that short-chain fatty acids regulate host gene expression by modulating miRNAs implicated in intestinal homeostasis and malignant transformation. An additional study by Humphreys et al. (2012) explored the effects of several histone deacetylase inhibitors (HDI) on miRNA expression in human colon cancer cell lines (HCT116 and HT-29). They reported that these HDIs also decreased miR-17~92 cluster miRNAs, while their target genes, e.g., PTEN, BCL2L11, CDKN1A, were increased. When miR-17~92 cluster miRNAs were overexpressed in the presence of HDIs, the protective effects of HDIs were diminished. Similarly, Wolter and Stein (2002) showed that resveratrol intensified the differentiation-inducing effects of butyrate in colorectal cancer cells. We observed that when dietary n-3 PUFAs were combined with fermentable fiber (pectin) in carcinogen injected rats, it led to the increased expression of miR-19b, miR-26b, miR-27b, miR-200c, and miR-203 and decreased the expression of their predicted targets, some of which have been shown to mediate oncogenic signaling. Collectively, these findings support the claim that pleiotropic bioactive components generated by fermentable fiber (butyrate) and fish oil (DHA and EPA) work coordinately to protect against colon tumorigenesis (Shah et al., 2011).

### ROLE OF VITAMINS IN miRNA MODULATION

#### Vitamin A

All-trans-retinoic acid, the most biologically active metabolite of vitamin A, is an essential dietary factor involved in vision, cell growth and differentiation, and immune function and acts as a tumor suppressor in lung, liver, bladder, prostate, breast, and pancreatic cancer models (Sun et al., 2002). In two separate studies using acute promyelocytic leukemia cells, retinoic acid exposure up-regulated miR-186, miR-215, miR-223 (Rossi et al., 2010), miR-15a, miR-15b, miR-16-1, let-7a-3, let-7c, let-7d, miR-107, miR-223, and miR-342 (Garzon et al., 2007) and down-regulated miR-17, miR-25, miR-93, miR-193, and miR-181b. In breast cancer (MCF-7) cells, retinoic acid exposure inhibited cell proliferation by modulating miR-21 (Terao et al., 2011). More detailed studies need to be carried out to elucidate how retinoic acid modulates miRNA levels and whether this phenomenon is responsible for its chemoprotective properties.

#### Folic acid

Folic acid is converted to 5-methyltetrahydrofolate and is abundant in fruits, vegetables, and grains. It serves an important role in DNA synthesis, repair, and methylation. Several studies have demonstrated the modulation of miRNAs by folate in a number of model systems. For example, when male Fisher rats were fed a folate-deficient diet, they developed HCC at 54 weeks in the absence of carcinogen. The onset of cancer was associated with the up-regulation of several miRNAs, such as let-7a, miR-21, miR-23, miR-130, miR-190, miR-17-92 and the down-regulation of miR-122 in liver tumors as compared to rats receiving adequate folate. After 36 weeks of folate replenishment, miR-122 levels were increased and associated with the inhibition of liver tumorigenesis. These findings indicate that a chemoprevention paradigm which involves folate affects miRNAs (Kutay et al., 2006; Pogribny et al., 2008). In human lymphoblastoid cells, folate deficiency produced a pronounced global increase in miRNA expression, including miR-222 (Marsit et al., 2006). These studies demonstrate that dietary modulation of miRNA expression is reversible.

#### Vitamin D

Clinical and epidemiological studies have shown that vitamin D (calciferol) and its metabolites 1,25-dihydroxyvitamin D3 (1,25(OH)_2_ D_3_) and 25-hydroxyvitamin D3 (25(OH)D_3_), exert protective effects by inducing G0/G1 arrest, cell differentiation, apoptosis, via modulation of a range of signaling pathways (Garland et al., 2011; Fleet et al., 2012). The classical model of action of 1,25(OH)_2_ D_3_ is via the vitamin D receptor (Fleet, 2004). Recent studies have suggested that vitamin D may exert its protective effects by modulating miRNA expression and its targets. Specifically, in human myeloid leukemia cells, vitamin D3 down-regulated miR-181a and miR-181b, resulting in an up-regulation of p27^KIP1^ and p21^CIP1^ and cell cycle arrest (Wang et al., 2009b). Additionally, vitamin D treatment up-regulated miR-32, which was associated with the inhibition of Bim and AraC-induced apoptosis (Gocek et al., 2011). Mohri et al. (2009) observed that miR-125b modulated the expression of the vitamin D receptor, through which the cancer chemoprotective effects of vitamin D are mediated. In malignant melanoma cells, Essa et al. (2010) observed an inverse relationship between miR-125b expression and vitamin D3 receptor levels. In colon cancer cell lines (SW480-ADH and HCT116), expression of miR-22 was induced by 1,25(OH)_2_D_3_ and when miR-22 was inhibited, the anti-proliferative and anti-migratory effect of 1,25(OH)_2_D_3_ was suppressed. Bioinformatic analysis demonstrated that genes affected by 1,25(OH)_2_D_3_ are also predicted targets of miR-22. Also, in human colon tumors, reduced expression of miR-22 correlated with vitamin D receptor expression as compared to the matched normal tissue. These data help to explain the mechanism of action of vitamin D and how it modulates gene expression via changes in miRNA synthesis/degradation (Alvarez-Diaz et al., 2012).

### MODULATION OF miRNAs BY PHYTOCHEMICALS

#### Polyphenols

Polyphenols are ubiquitous secondary metabolites found in fruits and vegetables, whole grain cereals, and beverages, including tea, coffee, and wines. Several clinical, experimental, and epidemiological studies have suggested an inverse association between polyphenol-rich food consumption and the prevention of chronic diseases (Arts and Hollman, 2005; Scalbert et al., 2005; Schroeter et al., 2006; Spencer et al., 2008). From a mechanistic perspective, polyphenols including ellagitannins, flavanol-rich extracts, epigallocatechin-3-gallate, curcumin, and resveratrol appear to modulate several miRNAs and their targets in several cancer models. Some of these findings are discussed below.

An in-depth study carried out by Milenkovic et al. (2012) examining liver metabolism in apolipoprotein E-deficient mice demonstrated that upon dietary polyphenol supplementation at doses that are considered nutritionally achievable, cellular functions were modulated by changes in miRNA expression. Specifically, exposure to nine polyphenols – quercetin, hesperidin, narangin, anthocyanin, catechin, proanthocyanin, caffeic acid, ferulic acid, and curcumin – modulated five overlapping miRNAs, miR-30c, miR-291b-5p, miR-296-5p, miR-373, and miR-467b, suggesting a common mechanism of action. Joven et al. (2012) demonstrated that consumption of a high fat diet significantly increased the liver expression of miR-103 and miR-107, but did not cause any change in target gene PANK1 expression. Supplementation with polyphenols resulted in reduction in the expression of miR-103, miR-107, and liver-specific miR-122. Collectively, these studies suggest that polyphenolic micronutrients exert their preventive effects, in part, by modulating the expression of select miRNAs.

#### Curcumin

Curcumin, a flavonoid derived from rhizomes of *Curcuma longa*, is considered to be a strong antioxidant with anti-inflammatory properties (Kuo et al., 1996). A number of studies have demonstrated that curcumin has protective properties against several types of cancer by modifying gene expression (Lopez-Lazaro, 2008). Sun et al. (2008) have demonstrated that treatment of human pancreatic cancer cells with curcumin resulted in the significant up-regulation of eleven miRNAs and down-regulation of eighteen miRNAs. Of these, miR-22 was the most significantly up-regulated non-coding RNA and was associated with the suppression of Sp1 and estrogen receptor 1, while miR-199a* was the most significantly down-regulated miRNA. Curcumin and its synthetic analog, diflourinated curcumin (CDF), either alone or in combination, down-regulated miR-200 and miR-21 expression, inducing the up-regulation of its target, PTEN, in pancreatic tumor cells (Bao et al., 2011). In another study by the same group, curcumin reduced EZH2 expression and increased a panel of tumor suppressive miRNAs, specifically let-7 family members, miR-26a, miR-101, miR-146a, miR-200b, and miR-200c (Bao et al., 2012). These data suggest that CDF inhibits pancreatic cancer tumor growth by targeting an EZH2-miRNA regulatory circuit. Soubani et al. (2012) also assessed the effects of CDF and BioResponse 3,3^′^-diindolylmethane (BR-DIM; a natural derivative of curcumin) on pancreatic cancer cells. The treatment increased levels of miR-200 and PTEN, while reducing the expression of MT1-MMP. This is noteworthy, because the loss of miR-200 and PTEN expression is a causative factor linked to the aggressive behavior of pancreatic cancer cells. Recently, curcumin was implicated in the reduction of WT1, a transcription factor, in pancreatic cancer cells (PANC-1) and leukemia cells (K562 and HL-60) (Glienke et al., 2009; Gao et al., 2012). Its effects were linked to the down-regulation of miR-15a/16-1. Hence, curcumin analogs may have application in the treatment of pancreatic cancer (Zhang et al., 2010).

With respect to other forms of cancer, e.g., breast cancer cells (MCF-7, SKBR-3, and Bcap-37), curcumin reduced the expression of Bcl2 by up-regulating miR-15a and miR-15b (Yang et al., 2010). In colorectal cancer cells, curcumin inhibited the transcriptional regulation of miR-21 via AP-1 and also suppressed cell proliferation, tumor growth, invasion, and *in vivo* metastasis (Mudduluru et al., 2011). In addition, curcumin has been shown to promote apoptosis in A549/DDP multidrug-resistant human lung adenocarcinoma cells by its action on miR-186*. Collectively, these data suggest that modulation of miRNA expression may be an important mechanism underlying the biological effects of curcumin and the effects likely vary depending on the target tissue.

### MODULATION OF miRNAs BY RESVERATROL AND ITS ANALOGS

Resveratrol, a stilbenoid found in the skin of fruits, especially grapes, has protective properties against cancer in terms of its ability to modulate canonical signal transduction pathways that control cell division and growth, apoptosis, inflammation, angiogenesis, and metastasis (Jang et al., 1997; Bhat and Pezzuto, 2002; Latruffe et al., 2002; Delmas et al., 2006; Pallas et al., 2009). Recently, several studies have demonstrated that resveratrol may exhibit these protective effects at least in part by modulating miRNAs. Tili et al. (2010b) reported that upon treatment of colorectal cancer cells (SW480) with resveratrol, miR-663 was up-regulated, which was linked to the inhibition of TGF-β. In addition, miR-17, miR-21, miR-25, miR-92a, and miR-196a were down-regulated and their targets, PDCD4, PTEN, and Dicer, were reciprocally up-regulated. Also, resveratrol treatment of monocytic cells induced miR-663-dependent effects by targeting AP-1 through JunB and JunD and impaired the up-regulation of well-known oncogenic miRNA, miR-155 (Tili et al., 2010a). Moreover, in lung cancer and nasopharyngeal carcinoma cells, up-regulation of miR-663 was shown to promote cell proliferation via the TGF-β and p21 pathways. In human lung cancer cells (A549), resveratrol treatment led to a significant up-regulation of miR-194, miR-299, miR-338, miR-582, and miR-758 and down-regulation of miR-92a. The predicted targets of these miRNAs modulate apoptosis, cell cycle regulation, cell proliferation and differentiation (Bae et al., 2011). In human bronchial epithelial cells (16HBE-T), miR-622 was up-regulated following resveratrol treatment, which was associated with the inhibition of cell proliferation and suppression of 16HBE-T cell primary tumor growth in nude mice (Han et al., 2012). Also in lung cancer cells, Hu et al. (2012a) showed that treatment with resveratrol inhibited cell mobility through induction of mesenchymal-epithelial transition (EMT) and the overexpression of miR-520h, which in turn reduced FOXC2. Resveratrol treatment in prostate cancer cells down-regulated the oncogenic miR-17~92 and miR-106b clusters and up-regulated several miRNAs, including miR-150, miR-149, and miR-1290. Also, PTEN, which is a predicted target of some of these miRNAs, was up-regulated (Dhar et al., 2011). Hence, the discovery that resveratrol can modulate the levels of miRNAs by targeting pro-inflammatory and/or pro-tumorigenic factors provides a rationale to optimize resveratrol-targeted treatments for the purpose of manipulating the levels of critical miRNAs.

### MODULATION OF miRNAs BY CATECHINS

Epigallocatechin-3-gallate (EGCG) and other tea polyphenols have been shown to alter cancer growth by targeting key oncogenic signaling pathways (Mukhtar and Ahmad, 1999; Tachibana, 2009). EGCG exposure has been linked to apoptosis, NFκ-B activation, suppression of nitric oxide synthase, and up- or down-regulation of tumor suppressor genes/oncogenes such as MAPK and PKC (Surh et al., 2005). Tsang et al. (2010) observed that by treating HCC cells (HepG2) with EGCG, expression of 61 miRNAs including miR-16, let-7c, miR-18, miR-25, and miR-92 were up-regulated, while miR-129, miR-196, miR-200, miR-342, and miR-526 were down-regulated. Also, the pro-survival gene, Bcl2, was shown to be targeted by miR-16. Similarly, miR-30b was down-regulated in the same model following EGCG treatment (Arola-Arnal and Blade, 2011). In human and mouse lung cancer cells, the tumor suppressor effects of EGCG treatment were linked to miR-210 expression and the modulation of the hypoxia-inducible factor 1α (HIF-1α) pathway (Wang et al., 2011). In addition, in mouse prostate cancer cells, EGCG treatment resulted in the reduced expression of miR-21 and miR-330 (Siddiqui et al., 2011). In comparison, treatment with polyphenon-60 (green tea extract) in breast cancer cells (MCF-7) down-regulated oncogenic miRNAs miR-21 and miR-27 (Fix et al., 2010). These emerging data suggest that EGCG may inhibit cancer cell growth by targeting specific miRNAs.

### MODULATION OF miRNAs BY ISOFLAVONES

Soy isoflavones such as daidzein, genistein, and glycitein have been reported to have anti-carcinogenic effects, e.g., inhibition of cell growth, invasion, and metastasis (Barnes, 1997; Dixon and Pasinetti, 2010; Li et al., 2011). Recently, two studies have demonstrated that isoflavones are capable of modulating miRNA expression in pancreatic cancer. Li et al. (2009) showed that genistein treatment in pancreatic cancer cells resulted in the up-regulation of miR-200, which was associated with the down-regulation of validated targets ZEB1 (zinc finger E-box-binding homeobox 1), slug, and vimentin, known to play a role in epithelial mesenchymal transition. Also, induction of let-7 and inhibition of cancer cell growth was noted after genistein treatment in this model. With respect to pancreatic cancer cells, genistein treatment resulted in an up-regulation of miR-146a expression and the concomitant down-regulation of several oncogenic targets such as EGFR, MTA-2, IRAK-1, and NF-κB, consistent with the inhibition of pancreatic cancer cell invasion (Li et al., 2010). Chen et al. (2011) used prostate cancer cell lines to demonstrate that genistein up-regulated ARHI, a tumor suppressor gene, via down-regulation of miR-221 and miR-222. In addition, to determine the effects of genistein in ovarian cancer cells (UL-3A, UL-313), a global miRNA profiling study was carried out. The expression of 53 miRNAs was associated with the up-regulation of estrogen receptor α and β levels (Parker et al., 2009). In comparison, in human uveal melanoma cells (C918), genistein treatment reduced miR-27a levels and inhibited tumor growth in nude mice (Sun et al., 2009b). Incubation of hepatoma (HepG2) cells with isoflavone (an anthocyanin) down-regulated miR-20b and up-regulated miR-197, miR-532, and miR-1224 (Arola-Arnal and Blade, 2011). Additional studies are needed in order to determine the biological significance of these observations.

### MODULATION OF miRNAs BY INDOLES

Indole-containing compounds, such as indole-3-carbinol (I3C) and sulforaphane isolated from cruciferous vegetables have been reported to possess chemoprotective properties (Higdon et al., 2007). Recently, in breast, lung, and pancreatic cancer cells, these agents have been shown to modulate miRNA expression. Jin (2011) showed that treatment of MCF-7 and MDA-MB-468 breast cancer cell lines with 3,3^′^-diindolylmethane (DIM), an *in vivo* dimeric product of I3C, increased miR-21 expression and reduced expression of its target Cdc25A, consistent with a dose-dependent inhibition of cell proliferation and development of breast tumors in an *in vivo* xenograft model. In several human pancreatic cell lines, DIM treatment up-regulated let-7b, let-7c, let-7d, let-7e, miR-200b, and miR-200c. These pancreatic cells displayed EMT characteristics by down-regulating E-cadherin, vimentin, and ZEB1, and treatment with DIM-inhibited cancer cell growth (Li et al., 2009). Therefore, induction of miR-200 and let-7 by isoflavone could be important for designing novel therapies for cancers. Melkamu et al. (2010) observed that in lung tissues obtained from mice upon treatment with vinyl carbamate (a potent carcinogen causing lung tumors) and given I3C in the diet, the expression of several “oncomiRs,” miR-21, miR-31, miR-130a, and miR-146, were reduced as compared to mice injected with carcinogen in the absence of I3C. These results indicate that I3C is able to reduce the effect of carcinogens in the lung by modulating expression of key miRNAs. In comparison, in rats exposed to cigarette smoke, I3C treatment restored the expression of miR-34b, miR-26a, miR-125a, and miR-10a (Izzotti et al., 2010; Melkamu et al., 2010). Collectively, these preliminary results suggest that I3C and DIM could function as miRNA regulators in a number of cancer cell types due to their chemoprotective properties.

### MODULATION OF miRNAs BY ISOTHIOCYNATES

Another compound found in cruciferous vegetables, phenethyl isothiocyanate (PEITC), has been shown to modulate carcinogen metabolism in different tissues (Pappa et al., 2006; Higdon et al., 2007; Clarke et al., 2008; D’Agostini et al., 2009). Studies have demonstrated that PEITC modulates miRNA expression in lung and liver tissues. Izzotti et al. (2010) conducted two PEITC feeding studies in mice exposed to cigarette smoke and carried out miRNA profiling in lung and liver tissues. In mice exposed to cigarette smoke, PEITC counteracted the biological effect of cigarette smoke by modulating ten miRNAs, e.g., let-7a, miR-26a, miR-31, miR-125b, miR-135, miR-200a, and miR-382 in the lung, whereas mixed alterations were observed in the liver. These data suggest that PEITC protects the lung from cigarette smoke-induced miRNA alterations, but had different effects in the liver. This could be due to the presence of different cell types in the two organ systems. Hence, it is very critical to study the effects of such chemoprotective agents in several organ systems and not just the target system.

## CONCLUSIONS AND FUTURE PERSPECTIVES

**FIGURE 2:**
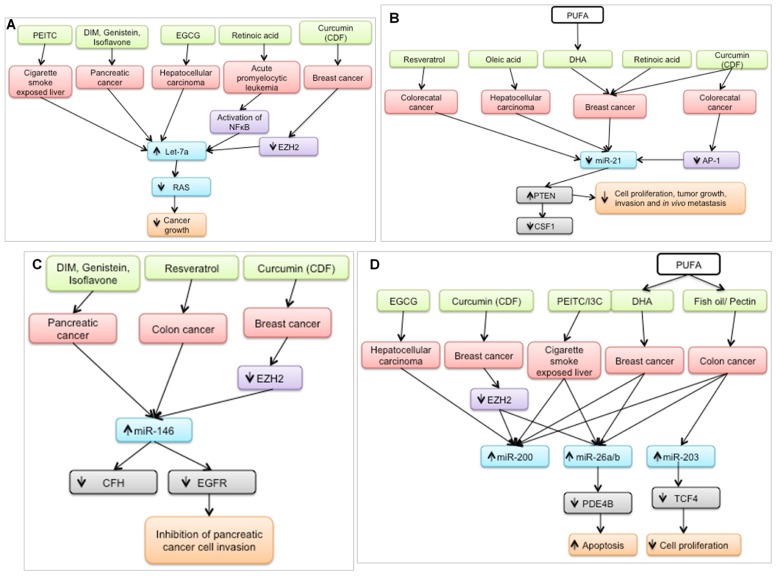
**Bioactive dietary agents modulate “oncogenic” miRNAs**. **(A)** let-7a, typically down-regulated in several types of cancer, is up-regulated by several chemoprotective dietary agents. Subsequently, RAS (gene target) expression is suppressed which coincides with a decrease in cancer growth. **(B)** miR-21, a well-defined oncogene, is down-regulated by several dietary agents in different cancer cell types, resulting in the up-regulation of one of its targets, PTEN, a well-known tumor suppressor. **(C)** miR-146 is up-regulated by chemoprotective diets resulting in down-regulation of its targets, leading to inhibition of cancer cell invasion **(D)** miR-200, miR-26a/b, and miR-203 are up-regulated by chemoprotective diets resulting in down-regulation of their respective targets, leading to increased apoptosis and decreased cell proliferation. PEITC, phenethyl isothiocyanate; DIM; diindolylmethane; EGCG, epigallocatechin gallate; DHA, docosahexaenoic acid, PUFA, polyunsaturated fatty acid, I3C, indole-3-carbinol.

In the last 5 years, a plethora of studies have examined the effect of nutritional bioactive agents on miRNAs and their targets in the context of cancer biology. Several of the targets of these miRNAs are tumor suppressors or oncogenes that mediate the initiation and progression of carcinogenesis. Examination of a broad range of miRNA studies involving dietary agents revealed that seven miRNAs – let-7a, miR-21, miR-26, miR-34, miR-125, miR-146, and miR-200 – were shown to be modulated by at least five agents as shown in **Figure 2**. It is possible that these miRNAs are preferred targets for chemoprotective dietary agents and may be used as indicators of the efficacy of dietary intervention.

A number of miRNAs exhibit complex trends of expression in response to dietary manipulation. This could be due to the fact that these miRNAs are expressed in a tissue-specific manner. For example, PEITC, a known bifunctional metabolic inducer, has been shown to exert different effects in lung and liver (Izzotti et al., 2010). Additional studies are needed to interpret the significance of these findings.

The majority of the studies with dietary agents have been performed in cancer cell lines. This is noteworthy because observations using cell line models are not always recapitulated *in vivo*. Clearly, *in vivo* whole animal studies are more likely to bear relevance to humans, especially since miRNAs are well conserved across species. Besides, *in vivo *approaches take into account the metabolic features of the bioactive dietary compounds. Also, most of the studies utilizing dietary agents are descriptive in nature. Hence, there is a need for in-depth examination of the temporal and functional mechanisms linking chemoprevention, miRNAs, and their target mRNAs. The majority of studies mentioned in this review have looked at the global effect of dietary bioactives on miRNA expression and only a few studies have validated the downstream targets. Additionally, it is necessary to probe the upstream mediators that are responsible for the alterations in miRNA expression. One of the causes of aberrant miRNA expression is the modification of histones and DNA methylation at the epigenetic level (Bao et al., 2004; Tuddenham et al., 2006). Some of the dietary agents such as butyrate, flavonoids, and curcumin are capable of altering the epigenetic landscape which can modulate gene/miRNA transcription and subsequently trigger changes in cell proliferation, differentiation, and cell survival (Fu and Kurzrock, 2010; Duthie, 2011; Berni Canani et al., 2012). Interestingly, several investigators have recently begun to explore how bioactive dietary agents alter the inter-regulatory patterns between promoter regions of miRNAs and several genes (Hu et al., 2011; Saini et al., 2011).

The interaction between bioactive dietary agents and SNPs in miRNAs (such as let-7a, miR-34, miR-125, miR-146, and miR-200) with respect to cancer risk is an open avenue of investigation. This may help improve our understanding of the inter-individual variability seen in response to dietary treatments. In addition, recent studies have shown that miRNAs in serum can serve as non-invasive biomarkers for cancer. Determining the change in miRNA levels in serum after exposure to dietary agents could be utilized as a diagnostic tool to monitor the effects of treatment over time. In addition, miRNA signatures could be used as potential biomarkers for cancer evaluation, once additional information regarding the role of miRNAs is obtained. Another major challenge for current miRNA studies is the need to identify the biologically relevant downstream targets that directly mediate the effect of the miRNA. The use of transgenic mice with a specific loss or gain of miRNA expression would help clarify the function of miRNAs and their targets *in vivo* (Rodriguez et al., 2007; Mu et al., 2009).

Results from a number of studies indicate that there is great interest to determine if combining conventional therapeutics with natural bioactive agents having chemoprotective properties is able to confer enhanced protection by modulating miRNAs and their targets. Because of the innocuous nature of dietary bioactives, it is likely that few, if any, safety concerns will arise. With respect to dietary molecular mechanisms of action, it would be worthwhile to determine how diet impacts components of the miRNA biogenesis pathway, specifically Dicer. Recently, it has been demonstrated that Dicer is a preferential cytoplasmic target for mutagens, which in turn affects miRNA maturation by competing with the pre-miRNA binding to Dicer (Ligorio et al., 2011). A recent study showed that dietary intake of natural products contributes to the prevention and treatment of diseases by regulating the miRNA biogenesis pathway (Hagiwara et al., 2012). It would be interesting to determine whether these dietary agents compete with mutagens and pre-miRNAs to affect the maturation of miRNAs. Insight from these studies will lead to a better understanding of the molecular mechanisms linking diet to chronic disease prevention.

## Conflict of Interest Statement

The authors declare that the research was conducted in the absence of any commercial or financial relationships that could be construed as a potential conflict of interest.
